# Fukushima simulation experiment: assessing the effects of chronic low-dose-rate internal ^137^Cs radiation exposure on litter size, sex ratio, and biokinetics in mice

**DOI:** 10.1093/jrr/rrv079

**Published:** 2016-01-29

**Authors:** Hiroo Nakajima, Yoshiaki Yamaguchi, Takashi Yoshimura, Manabu Fukumoto, Takeshi Todo

**Affiliations:** 1 Department of Radiation Biology and Medical Genetics, Graduate School of Medicine, Osaka University, 2-2, B4, Yamada-Oka, Suita, Osaka 565-0871, Japan; 2 Radioisotope Research Center, Osaka University, 2-4, B4, Yamada-Oka, Suita, Osaka 565-0871, Japan; 3 Department of Pathology, Institute of Development, Aging and Cancer, Tohoku University

**Keywords:** ^137^Cesium, chronic low-dose rate, internal radiation exposure, Fukushima simulation experiment, transgenerational effects, mice

## Abstract

To investigate the transgenerational effects of chronic low-dose-rate internal radiation exposure after the Fukushima Daiichi Nuclear Power Plant accident in Japan, 18 generations of mice were maintained in a radioisotope facility, with free access to drinking water containing ^137^CsCl (0 and 100 Bq/ml). The ^137^Cs distribution in the organs of the mice was measured after long-term *ad libitum* intake of the ^137^CsCl water. The litter size and the sex ratio of the group ingesting the ^137^Cs water were compared with those of the control group, for all 18 generations of mice. No significant difference was noted in the litter size or the sex ratio between the mice in the control group and those in the group ingesting the ^137^Cs water. The fixed internal exposure doses were ∼160 Bq/g and 80 Bq/g in the muscles and other organs, respectively.

## INTRODUCTION

The southern districts of Belarus are still highly radiocontaminated, even 28 years after the Chernobyl catastrophe of 1986. Similarly, the Fukushima Daiichi Nuclear Power Plant accident, caused by the tsunami of the Great East Japan Earthquake of 2011, has led to environmental changes that, in turn, have affected the soil, plants and animals. It is thought that radionuclides in the contaminated areas are being concentrated (through the food chain) in living organisms, and that they will remain in the irradiated organisms, both externally and internally, for long periods. Many scientists [1–4] have reported evidence of the effects of radiation on organisms in contaminated areas, e.g. the chromosome aberration rate in mice and frogs at Chernobyl was found to increase to levels that were twice as high as those before the catastrophe [1, 2]. However, most of the studies did not specify the exact level of radioactivity or the period of exposure. The exact amount of radioactivity in organisms needs to be known in order to assess the long-term low-dose rate and the low-dose internal and external radiation effects.

We measured the ^137^Cs radioactivity and its distribution in plants (trees and berries) and animals (insects, frogs, moles and mice) in a high-contamination area (Masani village, Gomel region, Belarus) and in a low-contamination area (Babchin village, Gomel region, Belarus) [5–7]. The ^137^Cs activity in mice in 2005 was compared with that in mice in 1997. The ecological half-life of ^137^Cs was estimated at 1.511 years [8], and ∼2% of the ^137^Cs was still present in the organs of the animals in 2005.

The health effects of radioactive contamination on residents of contaminated areas such as Belarus, Russia and the Ukraine have been studied in various ways during the 30 years since the Chernobyl Nuclear Power Plant accident. Among these studies, there have been reports of acute and late radiation sickness attributable to high-dose exposure of people involved in the decontamination and fire control efforts immediately after the accident [9, 10]. In addition, the exposure of children to radioactive iodine reportedly had an obvious effect on the occurrence of pediatric thyroid cancer [11, 12]. However, the various reports on the health effects on residents of radioactively contaminated areas (including various cancers, developmental deformities, and genetic effects) have yet to be confirmed.

^137^Cs, with a half-life of 30.2 years, is a major nuclide that persists in contaminated areas. Early reduction in radioactive contamination from natural purification could be expected, but the purification rate of ^137^Cs in undisturbed soil is relatively slow. In addition, internal exposure of organisms living in such environments, including humans, could be expected to continue for multiple generations, much longer than the initial external exposure. However, various studies pertaining to the Hiroshima and Nagasaki atomic bomb survivors have found no significant differences in genetic mutations between the exposed and the non-exposed groups [13–15]. Moreover, studies on the decontamination workers in Belarus and their children have also shown no significant next-generation effects thus far [16–18]. Because these investigations could only study the F_1_ generation, the presence or absence of accumulated transgenerational effects was unclear.

To simulate the radiocontamination in the low-contamination areas of Belarus and Fukushima (Japan), mice were maintained by brother–sister mating for seven years in the radioisotope facility, with free access to drinking water containing ^137^CsCl (0 Bq/ml and 100 Bq/ml). Subsequently, to simulate the Fukushima disaster, we monitored the litter size, sex ratio, and biokinetics in the descendant mice to assess the effects of low-dose internal ^137^Cs radiation exposure in every succeeding generation of mice.

## MATERIALS AND METHODS

### Breeding of mice

All brother–sister mated A/JJmcSlc mice (the first pair of mice was purchased from Japan SLC Inc., Hamamatsu, Japan) were maintained in a barrier unit, with light from 4 a.m. to 6 p.m. at 23 ± 1°C, and 50–70% humidity. The mice were fed a γ-beam-irradiated mouse diet CRF-1 (Charles River Japan, Kanagawa, Japan), together with acidified, chlorinated and filtered water (Millipore, Bedford, Massachusetts).

Two pairs of mice were selected from among the A/J strain littermates bred in the radioisotope facility. One pair was assigned to the control group and bred, while offered drinking water *ad libitum*; the other pair was administered a low-dose internal radiation exposure of ^137^CsCl water (100 Bq/ml). The harvested offspring from both pairs were sibling-mated for more than 10 generations, corresponding to a human generation turnover of ∼300 years. All mice born in the facility were given a serial number, and records were kept of the number of the generations, sex, date of birth and date of death. The sex ratio and the litter size of 18 generations of both the control group and the group ingesting the ^137^Cs water were analyzed from these records.

This animal study plan was approved by the Institutional Review Board of Osaka University. All animal experiments were performed at the Institute of Experimental Animal Sciences in accordance with the Osaka University Guidelines for Animal Experimentation.

### *In vivo* dynamics of ^137^Cs

To observe organ-specific temporal change in the ^137^Cs levels after single oral administering, ^137^CsCl water (100 Bq/g body weight) was administered to 2-month old male and female mice by stomach tube. Samples of the organs were collected at 0.5, 1, 3, 6, 9, 27, 54, 96, 192 and 389 h after administering the ^137^CsCl water.

The ^137^Cs distribution in each organ after long-term *ad libitum* intake of ^137^CsCl water (100Bq/ml) was measured in the F_0_ generation (at 1, 2, 4, 8, 14, 21, 29 and 57 d after starting the ^137^CsCl water supply) and in the F_18_ generation (male and female mice at 4 months old) and the fetus (pregnancy at 18 d).

The observation of the organ-specific temporal changes in the ^137^Cs levels of 9-month old female mice of the F_18_ generation, after the mice had started to drink non-radioactive water, was performed at 0, 3, 10, 16 and 34 d after starting the non-radioactive water supply.

### Sample collection

Mice maintained in the radioisotope facility were euthanized, and the organs (brain, heart, lung, liver, kidney, muscle, spleen, intestines, testes or ovaries, peripheral blood, and fetus) were excised, weighed and stored separately in plastic tubes.

### Radioactivity

The ^137^Cs gamma radioactivity (Bq per gram of wet weight) of mouse organs was measured by an automatic gamma counter 2480 WIZARD^2^ (PerkinElmer Co. Ltd, Waltham, MA, USA). The ^137^Cs gamma radioactivity of the entire mouse body was measured by a Ge semiconductor detector GC3018 (Canberra Inc., Meriden, CT, USA).

### Statistical analysis

The statistical analyses were carried out using SPSS (Ver. 6.1J) (SPSS Japan Inc., Tokyo, Japan) and KaleidaGraph software (Ver. 3.0.9) (Synergy Software, PA, USA).

More than three individual samples at a point were used for a *t*-test after analysis of the variance by an *F*-test.

## RESULTS

### *In vivo* dynamics of ^137^Cs

**Fig. 1.**
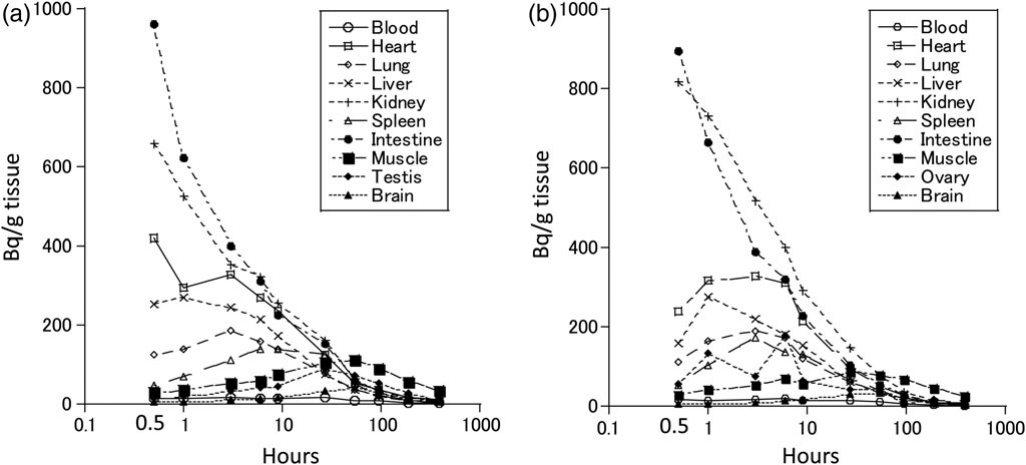
The organ-specific temporal changes in ^137^Cs levels after a single oral dose of ^137^CsCl water (100 Bq/g body weight) at two months of age. The ^137^Cs level of each organ was measured at 0.5, 1, 3, 6, 9, 27, 54, 96, 192 and 389 h after administering (each point; *n* = 1). (**a**) Male mice, (**b**) Female mice.

Figure 1 shows the organ-specific temporal changes in the ^137^Cs levels after a single oral dose of ^137^CsCl water (100 Bq/g body weight) in male (Fig. 1a) and female (Fig. 1b) mice, at 8 weeks of age. The half-life of ^137^Cs was short (within 2 d) in most of the organs (heart, lung, liver, kidney, spleen, intestines, testes or ovaries, and peripheral blood). However, in the muscle and brain tissue, the breakdown of ^137^Cs was delayed: the peak level occurred at 54 h in the muscle tissue of the male mice (111 Bq/g) and at 27 h in the muscle tissue of the female mice (86 Bq/g); the peak level occurred at 54 h in the brain tissue of both the male (41 Bq/g) and female mice (32 Bq/g), and subsequently decreased by half at approximately Day 6.

**Fig. 2.**
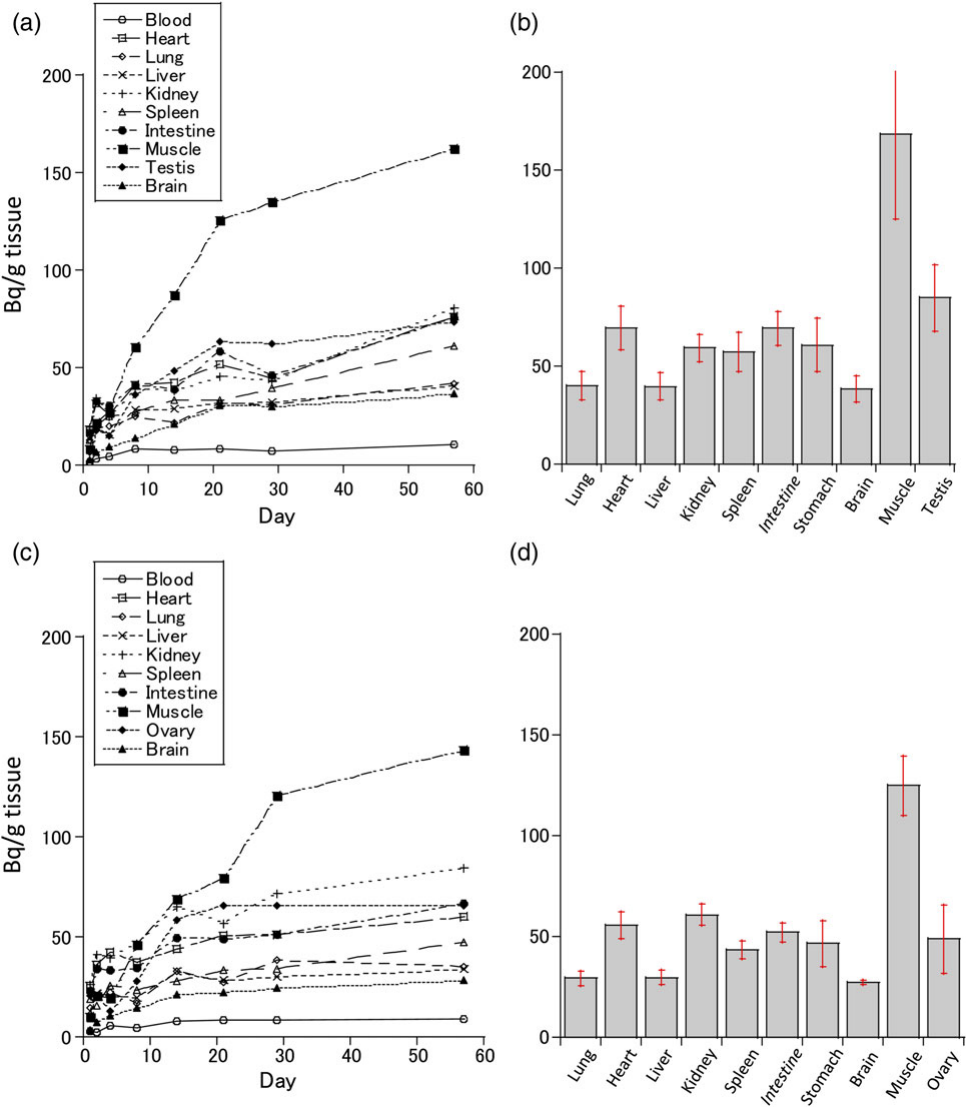
The accumulation of ^137^Cs over time in each organ in mice that had ingested water supplemented with ^137^CsCl (100 Bq/ml). The ^137^Cs level of each organ was measured at 1, 2, 4, 8, 14, 21, 29 and 57 d after starting the ^137^CsCl water supply. (**a**) Three- to five-month-old male mice in the F_0_ generation (each point *n* = 1), (**b**) four-month-old male mice in the 18th generation (*n* = 4, error bar represents 95% CI), (**c**) three- to five-month-old female mice in the F_0_ generation (each point *n* = 1), and (**d**) four-month-old female mice in the 18th generation (*n* = 4, error bar represents 95% CI).

Figure 2 shows the accumulation of ^137^Cs over time in each organ in male (Fig. 2a) and female (Fig. 2c) mice after continuous 2-month *ad libitum* ingestion of water supplemented with ^137^CsCl (100 Bq/ml). An average daily water consumption of 4.0 ml in males (3.1–4.8 ml) and 3.1 ml in females (2.8–3.5 ml) corresponded to 400 Bq and 320 Bq, respectively. The ^137^Cs intake per mouse per day averaged 16 Bq per 1 g body weight for a 25-g mouse (males) and a 20-g mouse (females). The accumulated level of ^137^Cs increased slowly to ∼10 times the average daily intake per 1 g body weight in the muscles by 8 weeks, while the levels in the other organs reached an equilibrium concentration less than half of that seen in the muscles.

Figure 2b and d show the accumulation of ^137^Cs over time in each organ/tissue (Bq/g tissue) in male (Fig. 2b) and female (Fig. 2d) mice at 4 months of age after 18 continuous generations and seven years of *ad libitum* intake of water supplemented with ^137^CsCl (100 Bq/ml). The mean whole-body ^137^Cs activities in males and females were 2313 Bq (93.5 Bq/g body weight) and 1158 Bq (56.4 Bq/g body weight), respectively. The level of ^137^Cs in the organs (lung, liver, intestines and brain) and whole bodies of male mice was significantly higher than in that of female mice (*P* < 0.05) (Fig. 2b and d). The levels of ^137^Cs in all the organs of the mice of the 18th generation were similar by Day 57 (Fig. 2a and c).

Additionally, activity in the fetus (pregnancy 18 d, *n* = 9), placenta, and amniotic fluid in the 18th generation was 15.1, 22.9 and 3.4 Bq/g, respectively.

The weight percentage mass of the body remaining after the excision of the brain, heart, lung, liver, kidney, spleen, intestines, and testes or ovaries in male and female mice was 57.6 and 53.6% of the intact body weight, respectively. The ‘empty’ bodies (what remained after the excision of the organs) were primarily composed of muscle tissue. Therefore, a much higher quantity of ^137^Cs radioactivity was present in the empty bodies (1785 Bq in males and 979 Bq in females) than in the excised organs.

**Fig. 3.**
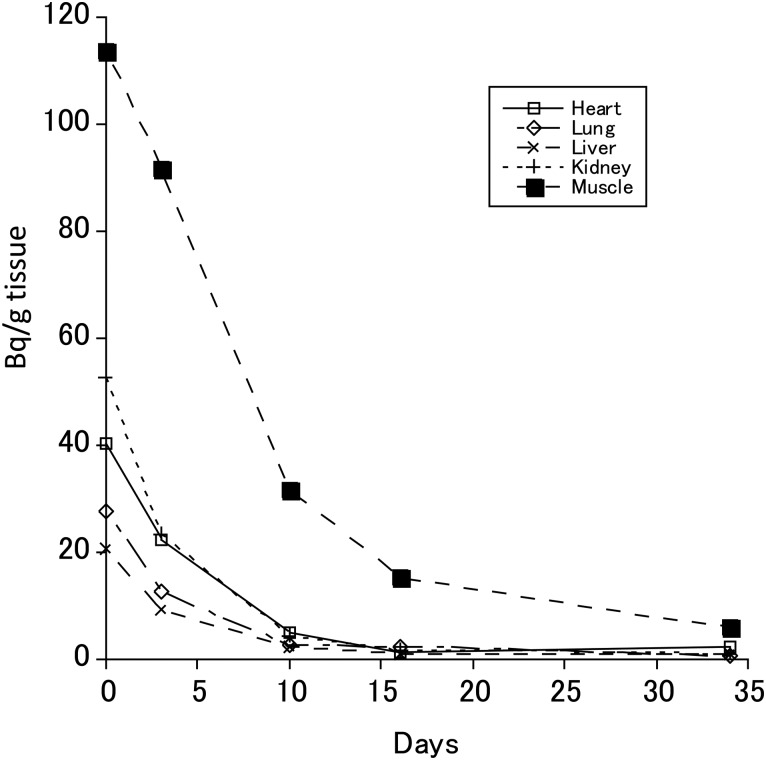
The decrease in the ^137^Cs level in eight-month-old female mice in the 18th generation after the mice had started to drink non-radioactive water. The ^137^Cs level of each organ was measured at 0, 3, 10, 16 and 34 d after commencing the supply of non-radioactive water. The whole-body ^137^Cs level at each point was 1542, 1213, 417, 202 and 79 Bq, respectively.

Figure 3 shows organ-specific temporal changes in the ^137^Cs levels of 8-month-old female mice of the 18th generation after the mice had commenced drinking non-radioactive water. The decrease in ^137^Cs level in the skeletal muscle was slower than in the myocardium, lung, liver and kidney. The estimated biological half-life of the ^137^Cs level in muscle, myocardium, lung, liver, kidney and the whole body was approximately 6, 4, 3, 3, 2.5 and 6 d, respectively.

### Sex ratio and litter size

**Fig. 4.**
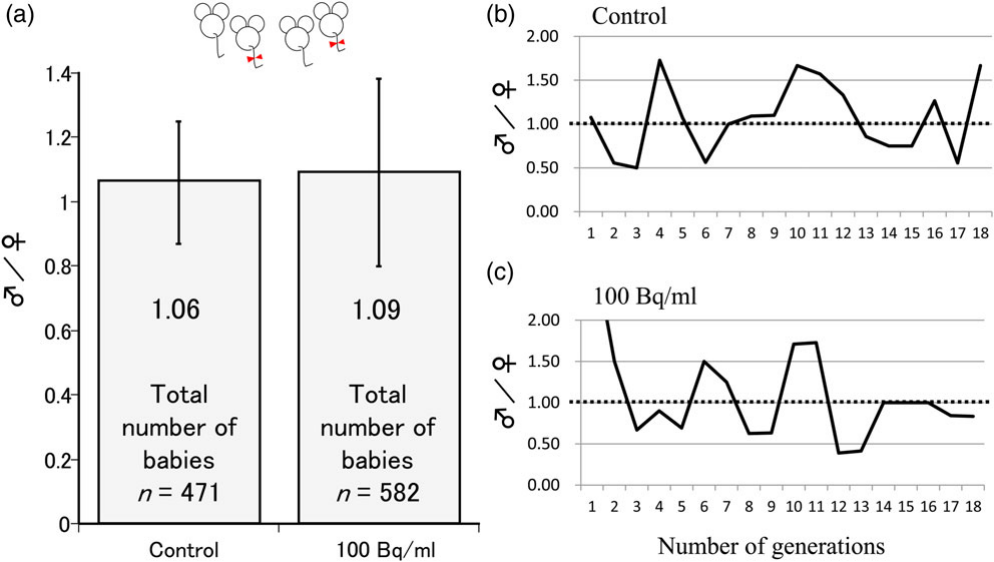
The sex ratio. (**a**) The mean sex ratio of all generations (F_1_–F_18_) (error bar represents 95% CI); (**b**, **c**) change in the mean sex ratio in each generation from F_1_ to F_18_.

The mean sex ratio of all the generations (F_1_–F_18_) is shown in Fig. 4a. The sex ratios of 471 babies in the control group and 582 babies in the group ingesting ^137^Cs water were 1.06 and 1.09, respectively. As shown in Fig. 4b and c, there was no change in the sex ratio from the F_1_ to the F_18_ generation.

**Fig. 5.**
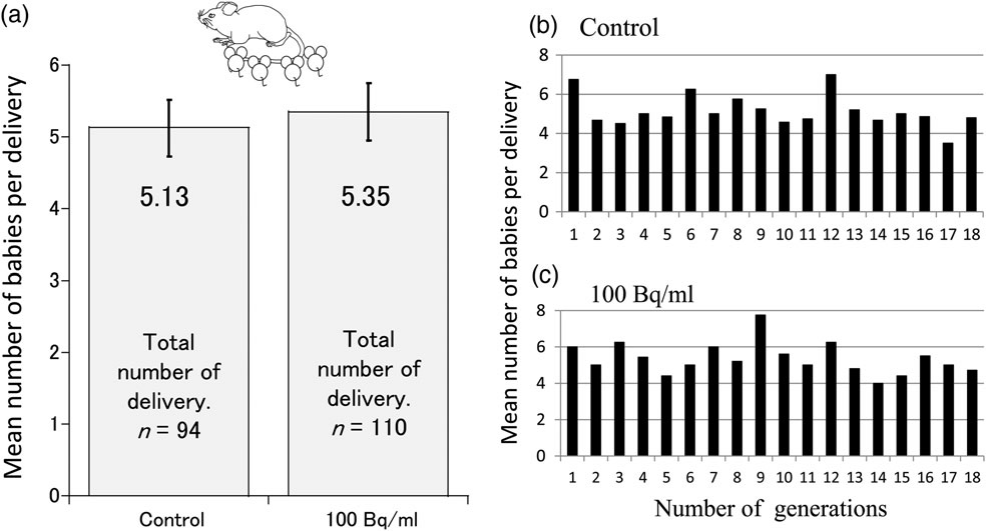
The litter size. (**a**) The mean litter size of all generations (F_1_–F_18_) (error bar represents 95% CI); (**b**, **c**) change in the mean litter size in each generation from F_1_ to F_18_.

The mean litter size of all the generations (F_1_–F_18_) is shown in Fig. 5a. The mean number of babies of 94 deliveries in the control group and 110 deliveries in the group ingesting ^137^Cs shows that there was no significant difference between the ^137^Cs-drinking group and the control group.

## DISCUSSION

The dynamics of ^137^Cs in organisms are similar to potassium dynamics. The muscle tissue had the highest amount of ^137^Cs contamination, and there were no distinct differences in the ^137^Cs distribution patterns in the body between the species measured (mice, mole and fish) in Belarus [6–8]. As shown in Fig. 2, the ^137^Cs distribution in the mice organs in the present study was similar to that indicated by our previous research on the radiocontaminated wild mice in Belarus [6–8]. Therefore, our experimental conditions could be accepted as reliably simulating the radiocontamination at Fukushima.

A single oral dose of ^137^Cs resulted in a peak muscle-tissue concentration similar to intake levels per 1 g body weight (Fig. 1). However, chronic and continuous dosing resulted in an accumulated concentration to ∼10 times the intake concentration per 1 g body weight (Fig. 2). Accordingly, if ^137^Cs was ingested continuously, the accumulated levels would not increase indefinitely, but would peak at a certain quantity. It is thought that the peak plateau of the ^137^Cs level depended on the organ/tissue and the intake amount; the ^137^Cs level in the organs decreased rapidly when the continuous intake of ^137^Cs ceased (Fig. 3). It appears that the speed of decrement of the ^137^Cs in the muscular tissue largely determined its speed of decrement in the whole body. Even when the daily intake of ^137^Cs (16 Bq per 1 g body weight) was similar in males and females, the body burden in males (93.5 Bq/g body weight) was significantly higher than that in females (56.4 Bq/g body weight). This difference could be due to the different retention times caused by body weight differences between males (24.8 g) and females (20.6 g). The difference could also be explained by the strong correlation between body weight and skeletal muscle weight, by the predominance of active transport in the cells of this tissue, and by muscle being slow-exchanging tissue [19].

Based on this observation, fixed internal exposure doses of ∼160 Bq/g and 80 Bq/g in the muscles and other organs, respectively, were continuously administered for 18 generations. The mean whole-body ^137^Cs activity at 3 months of age of males and females in the 18th generation was 2313 Bq (93.5 Bq/g body weight) and 1158 Bq (56.4 Bq/g body weight), respectively. The daily dose was estimated at ∼0.29 mGy (as measured only for internal radiation exposure by the beta particles) from the male mice whole-body ^137^Cs activity (93.5 Bq/g body weight).

However, it is suggested that the quantity of ^137^Cs in the body of the fetus (15.1 Bq/g) was less than the lowest quantity in the mother's organs (brain: 27.3 Bq/g) (Fig. 2d). In addition, the ^137^Cs level of the amniotic fluid (3.4 Bq/g) was less than half that in the mother's blood. The amount of ^137^Cs transmitted from the mother to the fetus thus appeared to be limited.

Therefore, the ^137^Cs level in mouse organs may not be constant over a single generation.

An increase in the number of female offspring of parents exposed to radiation (such as the survivors of the atomic bombing of Hiroshima and Nagasaki) could have been expected if such exposure had resulted in the induction of sex-linked lethal mutations. However, the data for the total number of births in Hiroshima and Nagasaki in the years from 1956 to 1962 indicate no such trend and no significant difference in the sex ratio in infants [20]. This result was obtained after observing one generation in a Hiroshima–Nagasaki study. We assessed the sex ratio and litter size in descendant mice (F_18_) exposed to low-dose internal ^137^Cs radiation exposure after every generation for 18 generations. No significant difference was noted in the sex ratio (Fig. 4) or the litter size (Fig. 5) between the individuals in the control group and those in the group ingesting ^137^Cs water over the 18 generations. This result suggests that low-dose and low-dose-rate internal ^137^Cs radiation exposure did not affect mouse litter size or sex ratio, a result that is similar to the results of the Hiroshima–Nagasaki study.

## CONCLUSION

Our experiment detailed the ^137^Cs biokinetics in mice, i.e. the litter size and sex ratio were not influenced by ^137^Cs ingestion, even after the mice had freely consumed cesium water (100 Bq/ml) over many generations. These results provide the basic data for our next series of experiments:

- detecting chromosomal abnormalities common to all cells by using multicolor fluorescence *in situ* hybridization (FISH),
- performing whole-genome sequencing to detect base sequence mutations in germ cells, which could accumulate in the non-coding regions in each generation,
- investigating the effects of low-dose radiation on carcinogenesis by quantifying the incidence and mean tumor volume of naturally occurring and urethane-induced lung tumors,
- detecting the effects of low-dose radiation on myocardium cell structures,
- metabolomic analysis of the heart and liver tissues of mice in the group ingesting ^137^Cs water and of mice in the control group, and
- detecting oxidative and anti-oxidative stress in the bodies of mice in the group ingesting ^137^Cs and in the control group.

Although novel discoveries are generally considered positive research findings, the negative results obtained in this study could contribute to the peace of mind of the inhabitants of radiocontaminated areas. The results of our Fukushima simulation experiments are considered informative, irrespective of whether biological effects were identified.

## FUNDING

Funding to pay the Open Access publication charges for this special issue was provided by the Grant-in-Aid from the Japan Society for the Promotion of Science (JSPS) [KAKENHI Grant No. 26253022].
